# External Validation of Risk Prediction Score for Candidemia in Critically Ill Patients: A Retrospective Observational Study

**DOI:** 10.3390/jof11030204

**Published:** 2025-03-06

**Authors:** Nurul Mazni Abdullah, Saw Kian Cheah, Raha Abdul Rahman, Nadia Md Nor, Muhammad Maaya, Qurratu Aini Musthafa

**Affiliations:** Department of Anesthesiology and Intensive Care, Hospital Canselor Tuanku Muhriz, Jalan Yaacob Latiff, Andar Tun Razak, Cheras, Kuala Lumpur 56000, Malaysia; nurulmazniabd@gmail.com (N.M.A.); raharahman1504@gmail.com (R.A.R.); nadiamn@hctm.ukm.edu.my (N.M.N.); muhammad@hctm.ukm.edu.my (M.M.); qurratuaini2112@gmail.com (Q.A.M.)

**Keywords:** candidemia, clinical prediction rule, intensive care, risk factors, acute kidney injury

## Abstract

Candidemia is associated with high mortality in critically ill patients. Early diagnosis of candidemia is imperative for starting treatment. Therefore, this study was designed to externally validate the candidemia risk prediction scores. This single-center, retrospective observational study included all critically ill patients admitted to the intensive care unit at a tertiary referral center from January 2018 to December 2023. The risks and patient outcomes were analyzed using bivariate and multivariate regression analysis. A total of 500 patients were analyzed with 2 dropouts due to incomplete data. Candidemia incidence was 8.86%, with parenteral nutrition and candida colonization identified as independent risk factors. Compared to an established risk prediction score, this study demonstrated a sensitivity of 75.0% [59.7–86.8], a specificity of 65.4% [60.8–69.8], a negative predictive value of 96.4% [94.2–97.8], and a positive predictive value of 17.3% [14.5–20.5]. The candidemia group had a significantly higher mean SOFA score, longer time in ICU, longer hospital length of stay, and higher rates of both ICU and in-hospital mortality. This study shows that the risk prediction score is more effective as a tool for excluding rather than predicting candidemia. We recommend against using it as the sole diagnostic guide.

## 1. Introduction

Candidemia is the presence of *Candida* species in the blood and is the fourth most common bloodstream infection in critically ill intensive care unit (ICU) patients. Invasive candidiasis (IC) comprises both candidemia and deep-seated *Candida* infection [1,2,3]. Candidemia is associated with high morbidity and mortality rates [2,3,4]. Studies have demonstrated that mortality is closely linked to both timing of therapy and/or source control [2,5]. To date, the main challenge in the management of candidemia is having an early diagnosis and recognizing criteria to start empirical treatment, especially in critically ill patients.

The epidemiology of candidemia in ICUs varies across regions and can be attributed to patient demographics, underlying risk factors, antifungal usage, and resistance patterns. In Europe, invasive candidemia incidence is 7.0 cases per 1000 ICU admissions, with an estimated crude mortality rate of 40%. *Candida albicans* is the leading species of candidemia in Europe, the United States, and Australia. There is a high prevalence of non-*albicans Candida* (naC) species in Asia and South America, driven by factors such as intrinsic azole resistance, biofilm formation, and high adaptive resistance potential. The growing concern of azole resistance in naC species and reported echinocandin resistance has been further complicated by the emergence of *Candida auris*, particularly evident in South Africa [1,2,4].

A direct blood fungal culture that enables susceptibility testing remains the gold standard for diagnosing candidemia [1,2,3,4,5]. However, the sensitivity of having positive fungal blood cultures is low and, often delayed as it needs prolonged incubation (>24 h) [4,5]. Use of biomarkers such as *Candida* antigen detection, mannan and anti-mannan antibodies, antibodies against *Candida* germinal tubes (CAGTA), 1,3-β-d-glucan, the detection of nucleic acids, and the T2 *Candida* nano diagnostic panel may produce faster results and can be considered as supporting evidence when culture results are not yet available in patients at high risk of candidemia. Unfortunately, despite their high sensitivity rate, these markers have their own limitations in them being used as sole confirmation tests [1,6]. Furthermore, these tests are not readily available in all institutions.

A few prediction scores have been proposed to risk stratify non-neutropenic critically ill patients that would benefit from early antifungal treatment and one of the recent ones was suggested by Jameran et al. in 2021 [3,5,7,8]. These scores include total parenteral nutrition (PN), recent surgical procedures, multifocal candida colonization, and severe sepsis as significant predictors for candidemia. Factors such as extremes of age, critical illness, the presence of a central venous catheter, antibiotic exposure, surgical intervention, immunosuppressive diseases, acute necrotizing pancreatitis, organ transplant recipients, candida colonization, and mechanical ventilation have also been suggested as risks for candidemia [1,2,3,4,5,6,7]. In 2016, the Infectious Diseases Society of America (IDSA) guidelines suggested that empirical antifungal therapy should be considered in critically ill patients with a high risk of candidemia when other causes of fever have been excluded [3]. However, this should be based on clinical assessment of risk factors, surrogate markers for invasive candidiasis, and/or culture data from non-sterile sites [3].

This study aimed to validate the candidemia risk prediction score by Jameran et al. [8] and also to determine the prevalence of candidemia and compare outcomes between candidemia and non-candidemia cases in our ICU patients.

## 2. Materials and Methods

This retrospective observational study received approval from the Research and Ethics Committee of the Department of Anaesthesiology & Intensive Care and the Medical Research & Ethics Committee of Hospital Canselor Tuanku Muhriz (HCTM) [JEP 2023-531]. This study included admissions of patients aged 12 years and above to the general ICU from January 2018 to December 2023. The age cut-off of 12 years was selected in accordance with our ICU admission policy, which sets the minimum age for admission to the adult ICU. Data were retrieved from the ICU registry. Demographic data, including age, gender, source of admission, i.e., emergency department (ED) or general ward, comorbidities, i.e., hypertension (HT), dyslipidemia, diabetes mellitus (DM), chronic kidney disease (CKD), malignancies, and autoimmune disease, patients’ sequential organ failure assessment (SOFA) score, requirement of vasopressors or inotropes, type of ventilator support, risk factors for candidemia according to the risk prediction score, length of stay (LOS) in the ICU and hospital, and ICU and in-hospital mortality, were documented. Patients with positive blood cultures with *Candida* sp. were grouped as candidemia.

**Figure 1:**
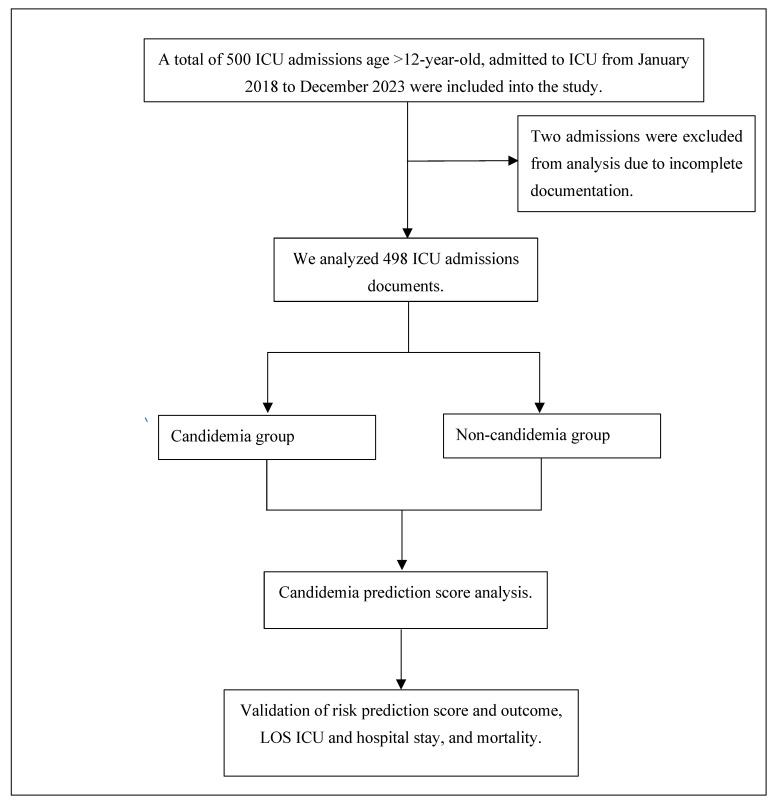
Flowchart of data inclusion.

All patients’ data were further evaluated against the risk factors for candidemia according to the risk prediction score by Jameran et al. [8]. Acute kidney injury (AKI), renal replacement therapy (RRT), multifocal *Candida* colonization, and PN were identified as independent risk factors for candidemia using multivariate logistic regression analysis. The assigned scores for each factor were as follows: AKI (2 points), RRT (4 points), PN (4 points), and multifocal *Candida* colonization (3 points) (Appendix A, Table A1). A total score of 5 or higher indicated a high likelihood of candidemia. Patients who were diagnosed with candidemia before their ICU admission or had positive blood cultures for *Candida* spp. within 48 h of ICU admission were excluded. Samples with incomplete documentation were also excluded from analysis. A flowchart illustrating data inclusion in this study is presented in Figure 1.

### Statistical Analysis

The sample size of this study was calculated using the Krejcie & Morgan formula for finite populations [9], based on the study by Jameran et al., which showed an incidence of candidemia of 3.27% in an ICU with 600 admissions per year [8]. Aiming for an 80% study power with a 95% confidence level, the sample size calculated was 450. After considering a 10% dropout rate, a total of 500 samples were collected. All data analyses were performed using SPSS for Windows version 27.0 (IBM Corp, Armonk, NY, USA). The Kolmogorov-Smirnov test was used to verify the normality of the distribution of continuous variables. Discrete variables were expressed as counts and percentages and continuous variables were expressed as the mean ± standard deviation or median (interquartile range) as appropriate. Continuous variables were analyzed using Student’s *t* test for parametric distribution and the Mann–Whitney U Test for nonparametric distribution. The discrete variable data analysis was performed using the Chi-Square test. A *p*-value < 0.05 was considered statistically significant. Factors with a *p*-value < 0.05 were analyzed for multivariate analysis. Sensitivity, specificity, positive prediction index (PPV), negative predictive value (NPV), positive likelihood ratio (PLR), and negative likelihood ratio (NLR) were calculated [10,11].

## 3. Results

This study included data from 500 ICU admissions. However, the analysis excluded data from two patients due to incomplete documentation. The ICU candidemia prevalence during the study period was 8.86% (88.6 per 1000 patients). Upon ICU admission, the demographic of both groups was comparable with an overall mean SOFA score of 6.77 ± 2.94. The bivariate analysis showed that mean SOFA score on admission to the ICU (OR: 1.15, 95% CI: 1.04–1.28, *p* = 0.006), the presence of AKI (OR: 2.99, 95% CI: 1.3–6.68, *p* = 0.010), the need for RRT (OR: 2.83, 95% CI: 1.51–5.31, *p* = 0.001), the use of PN (OR: 4.03, 95% CI: 1.38–11.77, *p* = 0.011), multifocal *Candida* colonization (OR: 4.22, 95% CI: 2.19–8.13, *p* < 0.001), and autoimmune diseases (OR: 3.11, 95% CI: 1.09–8.82, *p* = 0.03) (Table 1) were significant risks for candidemia. It was also shown that a significant percentage of patients in the candidemia group were on IPPV support (OR: 9.17, 95% CI: 2.19–38.42, *p* = 0.002). Concurrently, relatively higher percentages in the candidemia group (93.20%) than in the non-candidemia group (64.50%) required noradrenaline (OR: 7.51, 95% CI: 2.29–24.63). The overall median hospital stay was 15 [8–27] days and the ICU LOS was 6 [3–12] days. The candidemia group had a significantly greater number of ICU LOS of 12.00 [6.25–28.25] days (OR: 1.05, 95% CI: 1.02–1.07, *p* < 0.001) and hospital LOS of 31.50 [18.25–46.00] days (OR: 1.03, 95% CI: 1.02–1.04, *p* < 0.001). The in-hospital mortality rate for the candidemia group was higher (68.20%) than in the non-candidemia group (40.10%), *p* < 0.001. ICU mortality was also significantly higher in the candidemia group (43.18% vs. 28.85%, *p* = 0.48).

However, following further analysis using multivariate regression for the significant risks, only PN (OR: 3.20, 95% CI: 1.02–10.00) and *Candida* colonization (OR: 3.24. 95% CI: 1.59–6.60, *p* = 0.001) remained as independent risks for candidemia (Table 2).

In this study, a significant percentage of patients diagnosed with candidemia had a risk prediction score of ≥5, *p* < 0.001 (Table 3).

**Figure 2:**
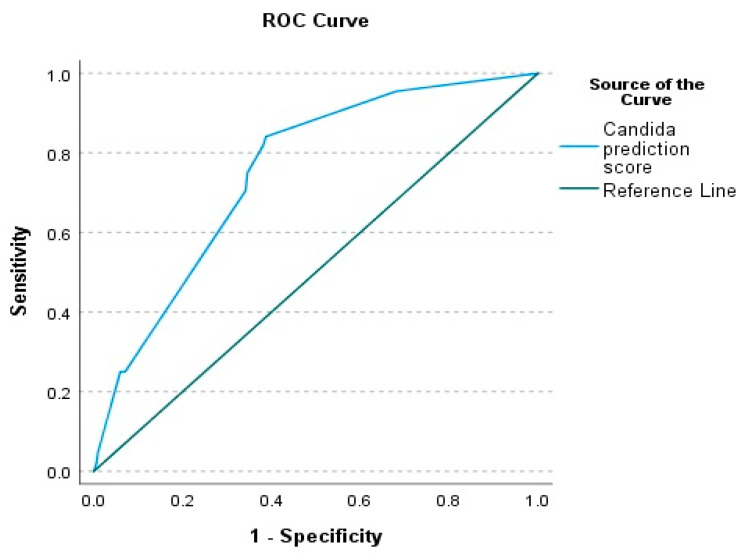
Receiver operator curve (ROC) for candidemia prediction score. The AUC of the ROC curve was 0.75 [95% CI: 0.68–0.82].

To discriminate the power of the risk prediction score, the area under the ROC curve was assessed as shown in Figure 2. The ROC curve analysis using the Youden index identified an optimal cut-off point of 2.50, achieving a sensitivity of 84.1% and specificity of 61.2%, with an AUC of 0.75 (95% CI: 0.68–0.82), a negative predictive value (NPV) of 97.6% [95.3–98.8], and a positive predictive value (PPV) of 17.3% [15.0–19.9]. The positive likelihood ratio (PLR) was 2.17 [1.82–2.57], while the negative likelihood ratio (NLR) was 0.26 [0.13–0.51] (Table 4).

In comparison, the risk prediction score with a cut-off point of 5.0 as recommended by Jameran et al. [8] demonstrated 75.0% [59.7–86.8] sensitivity and 65.4% [60.8–69.8] specificity, with a negative predictive value (NPV) of 96.4% [94.2–97.8] and a positive predictive value (PPV) of 17.3% [14.5–20.5]. The positive likelihood ratio (PLR) was 2.17 [1.75–2.68] while the negative likelihood ratio (NLR) was 0.38 [0.23–0.64].

The most common *Candida* species isolated were *Candida albicans* (34.09%)*,* followed by *Candida tropicalis* (27.27%), *Nakaseomyces glabratus* (15.91%)*,* and *Candida parapsilosis* (15.91%). Other fungal species isolated were *Tricosporon asahii* and *Curvularia* sp. The most common antifungal used was intravenous fluconazole (91.82%), followed by amphotericin B (10.91%), anidulafungin (2.75%), and voriconazole (0.91%).

## 4. Discussion

The candidemia risk prediction score was developed based on an observational study performed earlier in our ICU by Jameran et al. [8]. The prevalence of candidemia in our GICU has risen to 8.86%, which is more than double compared to earlier (3.72%). Others had reported lower incidence, ranging from 0.5–37.6 cases per 1000 [12,13]. Our ICU receives both medical and surgical cases, including patients with multiple organ failure, repetitive nosocomial infection, chronic illness, and malignancies. We also found that the mean SOFA score was higher in patients with candidemia. The proportion of patients who received IPPV and were on noradrenaline infusion as a vasopressor was also significantly higher. These findings may suggest that patients with candidemia were more ill compared to non-candidemia patients.

In this study, all of the factors included in the initial risk prediction score remained as significant risks, but not all were shown as an independent risk for candidemia. All of these risk factors have also been included in Paphitou’s rule and Ostrosky’s clinical prediction rule for candidemia [14,15,16]. AKI and the need for RRT are indicative of kidney failure. The presence of long-standing central catheterization and repeated use of external devices predispose patients to contamination and colonization of *Candida,* causing candidemia [14]. Colonization is believed to be a precursor to potentially invasive infections. Interestingly, in this cohort, only PN and *Candida* colonization were shown as significant independent predictive factors. However, we observed that up to three-quarters of patients diagnosed with candidemia had a risk prediction score of more than five. Mechanical ventilation, RRT, and other supportive treatments that involved breaches of protective barriers have been also included as factors to risk stratify candidemia [7,8,14,16].

Following that, we discriminated the power of the predictive score and found that the AUC of the ROC curve was 0.75 [95% CI: 0.68–0.82] with an optimal cut-off of 2.5, less than the initial score, which reported an AUC of 0.912 [95% CI: 0.867–0.956] with an optimal cut-off of 5.1 [8]. When performance was compared to the risk prediction score, it demonstrated a lower sensitivity of 75.0% vs. 80.3% and specificity of 65.43% vs. 77.3%, respectively. This study suggests that this predictive risk score was shown to be sensitive in excluding candidemia, as supported by the NPV and NLR values, but not in predicting candidemia, as the values of PPV and PLR were low. Similarly, the *Candida* colonization index (CCI) that was proposed by Pittet et al. showed a high NPV value of 96.9% [95% CI: 92.0–98.9], which suggests that the index is best in order to avoid unnecessary empirical antifungals, and it has a low PPV value, suggesting that empirical antifungals should not be started solely based on this test [17]. The *Candida* score, colonization index (CI), and Ostrosky’s clinical prediction rule (CPR) also showed a good NPV but a poor PPV and are useful for selecting patients who are not likely to benefit from antifungal therapies [18].

Although it has been reported that candidemia is not common in patients with systemic autoimmune diseases [19], interestingly, this study identified autoimmune disease as a significant risk for candidemia. Invasive fungal diseases in patients with autoimmune diseases are rare but have the potential to cause severe, opportunistic infections. However, *Pneumocystis jirovecii* pneumonia (PCP) is the most frequently identified fungal isolate [19,20].

Our result showed that patients with candidemia, when compared with the non-candidemia group, had a significantly longer hospital LOS of 31.5 vs. 14.0 median days and ICU LOS of 12.0 vs. 5.0 median days, respectively. This was consistent with a retrospective cohort study by Hohmann et al. with hospital LOS for candidemia of 42 median days (23.0–78.8) vs. the non-candidemia median of 8 days (5.0–7.0). The crude mortality for patients with candidemia in our ICU has been as high as 71.1% [8]. The mortality rate has decreased for both the in-hospital (68.20%) and ICU mortality (43.18%), as shown in this study despite the high prevalence. High mortality among candidemia patients has also been reported in other studies [14].

The incidence and distribution of *Candida* species varies within different regions with a reported naC is dominant, with *Candida parapsilosis* and *Candida tropicalis* being the most frequently isolated in Asia and South America [1]; however, in this study, the most common *Candida* species isolated were *Candida albicans*. We also isolated *Candida tropicalis*, *Nakaseomyces glabratus*, and *Candida parapsilosis*, which are known to be less sensitive to common antifungals. The most prescribed antifungal in our ICU was fluconazole, as we have limited access to anidulafungin and voriconazole. It is believed that the widespread use of fluconazole for prophylaxis and treatment can lead to the emergence of resistant *Candida* species.

This validation study was performed based on retrospective data in the same ICU where the initial predictive score was developed. Thus, these findings may only represent the local population. Therefore, prospective multicenter validation studies may need to be considered in the future.

## 5. Conclusions

Although the risk prediction score was shown to have good sensitivity with acceptable specificity, its NPV and NLR values suggested that this score was more useful as a tool to exclude rather than predict candidemia. Furthermore, only two of the risk prediction factors were shown as an independent risk. We agreed that the score was not robust enough to be used as a risk prediction score for candidemia and should not be used as a main diagnostic guide.

## Figures and Tables

**Table 1:** Demographic data, co-morbidities, and risk for candidemia are expressed as the number, n (%), mean ± SD, number (%), and median [IQR], as appropriate.

Variables	Non-Candidemia n = 454	Candidemia n = 44	*p*-Value	Odds Ratio	95% CI		*p*-Value
					Lower	Upper	
Age	59.70 ± 16.71	62.55 ± 16.20	0.28	1.01	0.99	1.03	0.27
Gender							
Male	273 (60.10)	25 (56.80)					
Female	181 (39.90)	19 (43.20)	0.67	1.14	0.61	2.14	0.67
Admission site							
ED	173 (38.10)	16 (36.40)					
Wards	281 (61.90)	28 (63.60)	0.82	1.08	0.57	2.05	0.82
Discipline							
Medical	339 (74.70)	29 (65.9)		Ref	Ref	Ref	0.52
Surgical	62 (13.70)	8 (18.20)		1.54	0.67	3.52	0.31
Orthopedics	25 (5.50)	5 (11.40)	0.34	2.35	0.84	6.58	0.11
Neurosurgical	16 (3.50)	2 (4.50)		1.47	0.32	6.69	0.62
Others	12 (2.60)	0 (0)		0	-	-	1.00
SOFA score	6.66 ± 2.95	7.93 ± 2.69	0.006	1.15	1.04	1.28	0.007
Hypertension	292 (64.30)	31 (70.50)	0.42	1.32	0.67	2.60	0.42
Diabetes mellitus	226 (49.80)	22 (50.00)	0.98	1.01	0.54	1.87	0.98
CKD	110 (24.20)	12 (27.30)	0.65	1.17	0.58	2.36	0.65
Dyslipidaemia	162 (35.70)	20 (45.50)	0.19	1.50	0.80	2.80	0.20
Malignancies	56 (12.30)	10 (22.70)	0.05	2.05	0.96	4.37	0.06
Respiratory diseases	61 (13.40)	9 (20.50)	0.25	1.66	0.76	3.62	0.21
HPB disease	22 (4.80)	1 (2.30)	0.71	0.48	0.06	3.65	0.48
Autoimmune disease	17 (3.70)	5 (11.40)	0.04	3.11	1.09	8.82	0.03 *
AKI	290 (63.90)	37 (84.10)	0.007	2.99	1.30	6.86	0.010
RRT	145 (31.90)	25 (56.80)	0.001	2.83	1.51	5.31	0.001
PN	15 (3.30)	5 (11.40)	0.024	4.03	1.38	11.77	0.011
*Candida* colonization	64 (14.10)	18 (40.90)	<0.001	4.22	2.19	8.13	<0.001
Ventilator support							
IPPV	316 (69.60)	42 (95.50)	<0.001	9.17	2.19	38.42	0.002
NIV	59 (13.00)	1 (2.30)	0.05	0.16	0.02	1.15	0.069
HFNC	55 (12.10)	0 (0)	0.01	0	-	-	0.997
Vasopressor use							
Noradrenaline	293 (64.50)	41 (93.20)	<0.001	7.51	2.29	24.63	<0.001
Adrenaline	53 (11.70)	5 (11.40)	1.00	0.99	0.37	2.62	0.986
Vasopressin	16 (3.50)	0 (0)	0.38	0	-	-	0.998
Dobutamine	33 (7.30)	3 (6.80)	1.00	0.93	0.27	3.18	0.912
Dopamine	12 (2.60)	1 (2.30)	1.00	0.79	0.10	6.18	0.821
Days of ICU stay	5.00 (3.00–11.00)	12.00 (6.25–28.25)	<0.001	1.05	1.02	1.07	<0.001
Days of hospital stay	14.00 (8.00–25.00)	31.50 (18.25–46.00)	<0.001	1.03	1.02	1.04	<0.001

Ref: reference; HPB disease: hepatobiliary disease; IPPV: intermittent positive pressure ventilation; NIV: non-invasive ventilation; HFNC: high-flow nasal cannula. A *p* < 0.05 was considered significant. * Not fit in the model using the Hosmer and Lemeshow fit test.

**Table 2:** Multivariate logistic regression of the risk factors for candidemia.

Variables	Odds Ratio	95% CI		*p*-Value
		Lower	Upper	
SOFA score	1.09	0.96	1.24	0.172
AKI	1.57	0.59	4.16	0.366
RRT	1.49	0.69	3.24	0.309
Parenteral nutrition	3.20	1.02	10.00	0.046 *
*Candida* colonization	3.69	1.86	7.30	<0.001 *

Variable(s) entered in the model: SOFA score, acute kidney injury, renal replacement therapy, parenteral nutrition, and *Candida* colonization. * *p* < 0.05 was considered significant.

**Table 3:** Candidemia risk prediction score vs. occurrence of candidemia.

Variables	Non-Candidemia Group n = 454, n (%)	Candidemia Group n = 44, n (%)	*p*-Value
Candidemia risk prediction score < 5	297 (65.40)	11 (25.00)	<0.001
Candidemia risk prediction score ≥ 5	157 (34.60)	33 (75.00)	

A candidemia risk prediction score ≥ 5 is predictive of candidemia occurrence.

**Table 4:** The cut-off value for the ROC curve.

Cut-Off Value	Sensitivity	Specificity
−1.00	1.000	1.000
1.00	0.955	0.681
2.50	0.841	0.388
3.50	0.818	0.381
4.50	0.750	0.346
5.50	0.705	0.341
7.00	0.250	0.070
8.50	0.250	0.066
9.50	0.250	0.059
11.00	0.045	0.009
12.50	0.023	0.007
14.00	0.000	0.000

## Data Availability

The datasets generated and analyzed during the current study are available from the corresponding author upon reasonable request.

## Appendix A

**Table A1:** Candidemia risk prediction score.

Risk Factors	Scores
Acute kidney injury (AKI)	2
Renal replacement therapy in the ICU	4
Parenteral nutrition	4
Multifocal *candida* colonization	3
Total score	

AKI is defined as when serum creatinine serum increase > 26.3 mmol/L within 48 h or 50% from baseline within 7 days or urine volume < 0.5 mL/kg/h for >6 h. Renal replacement therapy replaces nonendocrine kidney function through either intermittent hemodialysis, continuous hemofiltration (CVVH), or peritoneal dialysis (PD).

## References

[1] Noppè E., Eloff J.R.P., Keane S., Martin-Loeches I. (2024). A Narrative Review of Invasive Candidiasis in the Intensive Care Unit. Ther. Adv. Pulm. Crit. Care Med..

[2] Mora Carpio A.L., Climaco A. (2023). Fungemia Candidiasis.

[3] Pappas P.G., Kauffman C.A., Andes D.R., Clancy C.J., Marr K.A., Ostrosky-Zeichner L., Reboli A.C., Schuster M.G., Vazquez J.A., Walsh T.J. (2016). Clinical practice guidelines for the management of candidiasis: 2016 Update by the Infectious Diseases Society of America. Clin. Infect. Dis..

[4] Poissy J., Damonti L., Bignon A., Khanna N., Von Kietzell M., Boggian K., Neofytos D., Vuotto F., Coiteux V., Artru F. (2020). Risk factors for candidemia: A prospective matched case-control study. Crit. Care.

[5] Hohmann F.B., Chaves R.C.F., Olivato G.B., Souza G.M., Galindo V.B., Silva M.J., Martino M.D.V., de Menezes F.G., Corrêa T.D. (2023). Characteristics, risk factors, and outcomes of bloodstream *Candida* infections in the intensive care unit: A retrospective cohort study. J. Int. Med. Res..

[6] Bouza E., Almirante B., Rodriguez J.G., Garnacho-Montero J., Salavert M., Munoz P., Sanguinetti M. (2020). Biomarkers of fungal infection: Expert opinion on the current situation. Rev. Esp. Quimioter..

[7] León C., Ruiz-Santana S., Saavedra P., Almirante B., Nolla-Salas J., Álvarez-Lerma F., Garnacho-Montero J., León M.A., EPCAN Study Group (2006). A bedside scoring system (“*Candida* score”) for early antifungal treatment in nonneutropenic critically ill patients with *Candida* colonization. Crit. Care Med..

[8] Jameran A.S., Cheah S.K., Tzar M.N., Musthafa Q.A., Low H.J., Maaya M., Raha A.R. (2021). An approach to develop clinical prediction rule for candidemia in critically ill patients: A retrospective observational study. J. Crit. Care.

[9] Krejcie R.V., Morgan D.W. (1970). Determining sample size for research activities. Educ. Psychol. Meas..

[10] Unal I. (2017). Defining an Optimal Cut-Point Value in ROC Analysis: An Alternative Approach. Comput. Math. Methods Med..

[11] Diagnostic Test Evaluation Calculator MedCalc Software Ltd. https://www.medcalc.org/calc/diagnostic_test.php.

[12] Yapar N. (2014). Epidemiology and risk factors for invasive candidiasis. Ther. Clin. Risk Manag..

[13] Soulountsi V., Schizodimos T., Kotoulas S.C. (2021). Deciphering the epidemiology of invasive candidiasis in the intensive care unit: Is it possible?. Infection.

[14] Thomas-Rüddel D.O., Schlattmann P., Pletz M., Kurzai O., Bloos F. (2022). Risk factors for invasive *candida* infection in critically ill patients: A systematic review and meta-analysis. Chest.

[15] Hermsen E.D., Zapapas M.K., Maiefski M., Rupp M.E., Freifeld A.G., Kalil A.C. (2011). Validation and comparison of clinical prediction rules for invasive candidiasis in intensive care unit patients: A matched case-control study. Crit. Care.

[16] Ostrosky-Zeichner L. (2011). Clinical prediction rules for invasive candidiasis in the ICU: Ready for prime time?. Crit. Care.

[17] Pittet D., Monod M., Suter P.M., Frenk E., Auckenthaler R. (1994). *Candida* colonization and subsequent infections in critically ill surgical patients. Ann. Surg..

[18] Ahmed A., Baronia A.K., Azim A., Marak R.S., Yadav R., Sharma P., Gurjar M., Poddar B., Singh R.K. (2017). External validation of risk prediction scores for invasive candidiasis in a medical/surgical intensive care unit: An observational study. Indian J. Crit. Care Med..

[19] Vaquero-Herrero M.P., Ragozzino S., Iriart X., Castaño-Romero F., Sailler L., Sánchez-González R., Cassaing S., Charpentier E., Berry A., Carbonell C. (2020). Candida bloodstream infection in patients with systemic autoimmune diseases. Medecene Mal. Infect..

[20] Galmiche S., Thoreau B., Bretagne S., Alanio A., Paugam A., Letscher-Bru V., Cassaing S., Gangneux J.-P., Guegan H., Favennec L. (2023). Invasive fungal diseases in patients with autoimmune diseases: A case series from the French RESSIF network. RMD Open.

